# Iowa State Dental Society

**Published:** 1864-02

**Authors:** 


### IOWA STATE DENTAL SOCIETY.

The second meeting of the Iowa State Dental Society took place at Iowa City, January 20th, 1864, pursuant to call.

Neither the President or Vice-President being present, the Society was called to order by Dr. Kulp, of Muscatine; and, upon his nomination, Dr. N. H. Tulloss, of Iowa City (in whose office the meeting was held), was elected President *pro tem.* by acclamation.

Dr. McGarvey, Secretary, read the minutes of last meeting, which were approved. Also, a communication from our absent President, which was ordered to be placed on file.

On motion, the Secretary was requested to read the Constitution for the benefit of applicants for menbership.

On motion, new members were allowed to sign their names to the Constitution and Regulations of the Society; whereupon, six Dentists, from different parts of our State, embraced the previlege extended.

By vote of Society, Dr. E. M. Skinner, of Syracuse, N. Y. (who was present), was elected an honorary member of this Society.

On motion, Drs. Myers of Davenport, Coulson of Iowa City, and Smith of Tipton, were chosen to prepare subjects for discussion during the session.

Drs. Kulp of Muscatine, Robinson of Davenport, and Coulson of Iowa City, were appointed a committee on revision of Constitution; and ordered to report at next morning session.

The Society then took a recess for half an hour, to wait for a report from the committee on Subjects for Discussion.

The Society was again called to order, by the President.

The committee on Subjects for Discussion made the following report, which was accepted and the committee discharged:

1. Best mode and materials for filling teeth.
2. Treatment of diseased teeth.
3. Deciduous teeth and irregularities; and the best mode for regulating.

4. Mechanical dentistry, and vulcanite.
5. Anæsthetic agents.
6. Professional etiquette.
7. Dental fees.

1st. *Best mode and materials for filling teeth.*—Dr. Kulp of Muscatine was called on, who stated his manner of preparing cavities and filling teeth, with several materials. He preferred gold, in all cases where it could be used, and when patients were able and willing to pay for it. Felt very partial to sponge or crystal gold; could always build-out broken teeth to their original size and shape with it. Thought that a good amalgam filling was much better than a poor gold filling. He used amalgam considerably; took great pains in finishing up his amalgam fillings after they became hard; took same care in thoroughly clearing out cavities for amalgam fillings as he did with gold; thought that failure in this first step was, perhaps, the reason of most of the failures in amalgam fillings. Many dentists think it a cheap filling, and do the work cheap accordingly. This is all wrong; dentists should always do the very best possible for their patients, or not undertake the case.

Dr. Robinson, of Davenport, preferred gold in all cases where it could be used,—sponge gold, except in very small proximal cavities close to the gum, where he used soft gold-foil,—for the main reason, that it could be put in in less time than sponge or crystal gold, and thus avoid getting your filling wet, and fail, as he many times did when he tried to use sponge or crystal gold. Used some amalgam; thought with Dr. Kulp, that a good amalgam filling was better than a poor gold filling. Wherever he failed in saving teeth by filling, he most always found it was in cases where he failed to properly cleanse the cavity or condense and finish his fillings.

Dr. Kulp said that in such cases, where there was danger of getting the filling wet, either by the bleeding of the gums or from mucus contained around the necks of teeth, he found that by applying a little percloride of iron, and forcing a pledget of cotton upon the margin of the gum, there was no further trouble; and could use sponge gold with perfect success.

Dr. Harris, of Rock Island, Illinois, always recommended gold for filling teeth; used crystal and S. S. White's soft foils generally, did not use much crystal gold, thought it was not entirely free from acid; perhaps the present manufacture was purer than when he tried it; always condenses and finishes his fillings as perfectly as possible.

Dr. Smith, of Tipton, thought gold the best material for filling teeth, used only gold foil, never tried sponge gold, used crystal gold foil; does not use much amalgam, but uses a great deal of tin foil.

Dr. Myers, of Davenport, uses gold for filling teeth generally, sometimes a little amalgam or tin foil, uses crystal gold foil sometimes, but generally Abbey's soft foil.

Dr. Coulson, Iowa City, uses gold for filling teeth when he can get his pay for it; when not, he uses tinfoil, thought he would try amalgam.

2d. Dr. Robinson, of Davenport, used for destroying exposed pulps, creosote and arsenic, left in generally 24 hours; removed pulp and nerve as nearly as possible and put in cotton saturated with creosote and tannin, and directed patient to return in three or four days, when he filled tooth. Cured about three out of five teeth with diseased periosteum, treated with creosote and tannin generally.

Dr. Kulp,—treated exposed nerves in a similar manner to Dr. R., except that he did not fill the tooth for about ten days after removing the nerve; made broaches out of steel such as is used in "hoop skirts" for removing nerves. Was always sure of saving tooth with diseased periosteum when he could force creosote through the tooth and come out through opening of abscess; saved about half of such teeth; found the greatest difficulty with superior bicuspids.

Dr. Harris said he did not leave creosote and arsenic in the tooth longer than from six to eight hours; the bleeding he thought assisted in relieving or reducing inflammation, and thus considered it a benefit. He always removed pulp and nerve when possible, and after all soreness subsided he filled the tooth.

Dr. Smith, of Tipton, related a case of cure of alveolar abscess, and restoration to a healthy condition of tooth.

Dr. Skinner, of Syracuse, N. Y., was called upon. He gave a very minute description of his mode of treating diseased teeth, maintaining that a large per centage of such teeth could be saved—nearly, if not quite all, where nerve only was exposed.

All participated in a familiar discussion of various modes of treating such teeth, with benefit to all present.

Dr. Kulp finally gave a history of a severe case of alveolar abscess caused from a diseased central incisor; his manner of treating it with iodine, perchloride of iron, tannin and creosote, bringing it to a healthy state, and filling it with gold—since perfectly well. He thought the loss of half the teeth treated by the dentist, was attributable to the neglect of patients keeping their engagements—failing to return at the time appointed by the dentist. He urged dentists to try and educate their patients to be prompt in keeping their dental engagements, as he thought in thus training the public, we could save a great deal of pain and a great many more teeth.

On motion, adjourned until 9 A. M., January 21st.

SECOND DAY.

The meeting was called to order by the President, pursuant to adjournment.

Roll called, and minutes of preceding session read and approved.

Committee on revision of Constitution reported. Report was accepted, and Committee discharged.

On motion, Constitution was read and adopted article by article, and on recommendation of said Committee, the following order of business was adopted for the government of this Society at its future meetings.

1. Temporary organization of meeting preparatory to election of officers.
2. Calling roll by Secretary.
3. Reading minutes of last meeting.
4. Reception of new members and reading notes from absentees.
5. Reading reports of retiring officers.
6. Election of officers and their inauguration.
7. Reading and consideration of Reports of Standing Committees.
8. Reading of Essays.
9. Discussion of topics selected for session.
10. Selection of place for holding next meeting.
11. Appointment of Committees.
12. Resolutions introducing new business.
13. Appointment of Delegates to Dental Societies.
14. Unfinished and miscellaneous business.
15. Adjournment.

On motion, proceeded to elect officers for the ensuing term, which resulted in the following choice:

*President.*—Dr. N. H. Tulloss, of Iowa City.

*Vice-President.*—Dr. Wm. H. Robinson, Davenport.

*Secretary and Treasurer.*—Dr. A. J. McGarvey, DeWitt.

*Cor. Secretary.*—Dr. Wm. O. Kulp, of Muscatine.

Resumed regular subject for discussion.

3d. *Deciduous teeth, irregularities and best mode of regulating teeth.*—Dr. Robinson thought that deciduous teeth were generally prematurely extracted. We could not be too cautious in conclusions as to whether such should be taken out or not. To discuss the best mode of regulating irregularities was a hard subject, unless we had a subject in hand; it depended a great deal on the ingenuity of the dentist.

Dr. Newell, of Leclaire, thought that dentists as a general thing were too anxious to operate; he related a case in which he thought less operating would have proved beneficial to the patient. His opinion was that, the great majority of cases of irregularities were caused by premature extraction of teeth; the mode to regulate was to remedy this fault.

Dr. Myers, Davenport, has extracted a great many deciduous teeth, but only when in his opinion it was necessary—was aware that by premature extraction the alveolar arch would contract, and thus give less room for permanent set teeth. Did not have much trouble in correcting irregularities.

Dr. Harris thought that there were, no doubt other causes as potent in producing irregularities of teeth, but he would most emphatically condemn the wholesale extraction of deciduous teeth; the subject is not impressed on the minds of parents sufficiently by the dentist. Every dentist should consider himself a public teacher, and educate the community in which he practices on all these important subjects; thus his usefulness would be greatly augmented.

Dr. Kulp thought, as a general thing, neither parents or dentists consider the relative value of deciduous teeth as they should; dentists generally only looked after the fee for extraction; parents do not value their advice as they should. Many do not make an effort to *gain knowledge*, consequently their advice *costs* them *nothing*, and of course is freely given; it is worth *less than it costs*, and generally does a great deal of *mischief*. He related a case in his practice, of a little girl 10 years old, with cuspidatus and first bicuspids of lower jaw, entirely outside of the arch, a plain case of premature extraction of deciduous teeth.

4th. *Mechanical Dentistry—Vulcanite work.*—Dr. Robinson, of Davenport, used the Rubber extensively, liked it better than any other style of work. Did not use air chambers in full sets, and seldom in partial sets.

Dr. Myers, of Davenport, said he had used the "Vulcanite" for 18 months, and would condemn its general use for several reasons: 1st, It had been the cause of bringing the dental profession down to the standing of a mechanical trade, in prices at least. 2d, He saw several cases of severe inflammation of the mucous membrane of the mouth, where it had been worn, while he never saw but one case in all his practice in which gold produced this result; he was not prepared to say what caused this inflammation more in the use of Rubber than other materials. Did not use air chambers in all cases, but generally did.

Dr. Harris, of Rock Island, said he differed somewhat with Dr. M. on the Rubber question; he deemed it far superior to silver in all cases, and in many cases to gold; he thought that the dignity of the profession could be kept up by concerted action of dentists in their prices. He thought air chambers very essential in many cases, and generally used them.

Dr. Newell, of Laclaire, preferred Rubber for base for artificial teeth to every thing else except gold, and in some cases would not except that. In partial sets, used gold generally; discarded the use of air chambers, in partial as well as full sets.

Dr. Kulp used Rubber extensively, thought its introduction in the dental profession one of the greatest blessings of the age to the people. Dentists are generally apt to lose sight of the fact, that the object of our profession is to do good to the human family; thus any thing which will benefit the people should be highly prized by the dentist. Takes impressions for full sets, upper and lower, with plaster paris; for partial sets, with wax and parafine, such as is sold and prepared by S. S. White. Uses over models generally, "Barker's Ethereal Preparation," sometimes tin foil; thinks thickness of plate but little real objection, as a patient would no sooner get used to a covering of paper over palate, than to ¼ inch of Rubber, well polished. Makes partial lower sets in Rubber base altogether, and as to the inflammation referred to by Dr. Myers, he thought that it arose from too close fit over the gum, of plate, rather than from any deleterious matter in Rubber compound.

The various modes of getting up hard cast and swaging plates, were discussed at great length by all present. After which, Dr. Harris, of Rock Island, presented several beautiful specimens of work gotten up by him, after the style of what is known as "Dr. Fuller's Combination Vulcanite work." It is a continuous gum arch, gotten up in the usual style, and mounted in Vulcanite Rubber base. It is decidedly beautiful. If practicable, it will soon take a high stand in the profession. Dr. H's. testimony is, that he has seen no objections to it, having used it in his practice for some time.

A vote of thanks was tendered to Dr. Fuller, for his invention, (not his PATENT.)

Dr. Robinson said he admired the style of work, and hoped if it proved practicable and permanent, it would be used by all of us, and thus bring up the prices of artificial dentistry.

Dr. Myers said as regards Dr. F's. new style of work, I am glad Dr. Harris has presented it to this society at the present degraded condition of our profession, brought about by the use of Rubber. It is the duty of all to elevate the profession, and to do so, we must use such work as every quack can not get up, even if profits *are* smaller. This work looks better than any I ever saw, but can not speak for it *practically;* but will say, if we have to use Rubber, let it be by such combination that it may bring back what has been lost to the profession by its general use by quacks and others.

Dr. Harris thought that by introducing this style of work (which needed a greater amount of skill to get up than any other kind), we would debar quacks and humbugs from flourishing, and raise the price of dentistry and elevate the profession, and thus cause the public to pay more attention to their natural teeth, and not depend so much on getting a cheap set of teeth when old ones are gone.

On motion, adjourned until 2 P. M.

AFTERNOON SESSION.

Society called to order by the President, pursuant to adjournment.

Upon motion, the regular order of business was suspeneded in order to attend to other important matters.

Upon motion, the following Committees were then appointed:—Committee on Subjects for Discussion and Essays for next meeting—Drs. Coulson, Myers and Harris.

On Dental Fees—Drs. Myers and Robinson, of Davenport, and Dr. Harris, Rock Island, Illinois.

By a unanimous vote, Davenport was selected as the place, and the second Tuesday in August, at 8 P. M. as the time for holding the next meeting.

The following Resolution was offered by Dr. Robinson, which was adopted.

*Resolved*, That the dissemination of general dental knowledge would elevate the profession and benefit the public, and to gain this object the Society do hold public meetings at the usual times for regular meetings; and that one or more public lectures on some subject in Dentistry be delivered at such meetings; and that three members be appointed a committee to carry this resolution into effect.

Committee appointed—Drs. Robinson, Kulp and Chase.

Drs. Kulp and Minor were appointed a committee to take into consideration the propriety of publishing in local newspapers in our State, short Essays on Dentistry in general, for the express benefit of the public, and report at the next meeting.

Essayists appointed by Committee on Essays, for next meeting.

Diseases of the alveolus and treatment—Dr. Henry S. Chase, Independence, Iowa.

Deciduous teeth, care and treatment—Dr. J. Coulson, Iowa City, Iowa.

Exposed pulp, treatment preparatory to filling, etc.—Dr. Smith, of Tipton, Iowa.

The following Resolution was adopted:—

*Resolved*, That any member of this Society, attending any Local or National Dental Society, shall, upon presentation of Proceedings of this Society with his name in it, or a certificate from the Secretary showing his membership, shall act as Delegate for this Society.

On motion, Proceedings of this session of Society were ordered to be published in "Dental Cosmos," and "Dental Register of the West."

After a general expression of good feeling, and profit in thus coming together, promising to again be on hand at the August meeting, they adjourned until the second Tuesday in August next.

Dr. H. H. TULLOSS, *President.*

" WM. H. ROBINSON, *Vice-President.*

" A. J. MCGARVEY, *Recording Secretary.*

" WM. O. KULP, *Asst. Rec. Sec. & Cor. Sec.*

